# History of the Royal Academy of Sciences for the Year 1778; with the Mathematical and Philosophical Memoirs for That Year, Taken from the Registers of the Academy

**Published:** 1783

**Authors:** 


III. *Histoire de l'Academie Royale des Sciences. Année* 1778. *Avec les Memoires de Mathematique et de Physique, pour la meme année, tirés des Registres de cette Academie.* i. e. *History of the Royal Academy of Sciences for the year* 1778; *with the Mathematical and Philosophical Memoirs for that year, taken from the Registers of the Academy.* 4to. Paris, 1781. 707 pages, with twelve copper-plates.

THE first or historical part of this volume contains the eulogies of Paul James Malouin and the celebrated Linnæus, two members of the academy lately deceased.

Of M. Malouin, we are told, that he was born at Caen in Normandy in 1701. His father, who was a counsellor in that city, and designed him for the bar, sent him at a proper age to pursue his studies at Paris, and in 1730, when he returned to Caen, was surprized to find, that instead of being a graduate in civil law, his son had taken a doctor's degree in physic.

After

After ſpending about three years at Caen, he returned to Paris, where his chemical abilities procured him the friendſhip of M. Geoffroi.

M. de Fontenelle likewiſe, to whom he was nearly related, intereſted himſelf in his advancement, and after the death of M. Dumoulin he became one of the phyſicians the moſt employed in Paris. This celebrity laſted only twenty-two months; but at the end of that period he found himſelf rich enough to retire to Verſailles, where he died of apoplexy on the 3d of January 1778.

He was the author of a work on Medicinal Chemiſtry; of a treatiſe on the art of making bread, and of ſeveral chemical eſſays inſerted in the memoirs of the academy. All theſe writings have the character of being judicious, learned, and accurate.

No phyſician perhaps was ever more zealous for the intereſts of his profeſſion than M. Malouin. Several anecdotes in proof of this are related by his eulogiſt, and at his death, we are told, he founded a public meeting, which is to be held once a year by the College of Phyſicians at Paris for the purpoſe of doing honour to the memory of their deceaſed aſſociates, and as an incitement to medical improvement.

To the account we have already given of Linnæus (Vol. III. page 29) we may add from the volmue before us, that in compliance with the cuſtom of his country he exchanged the name of Linnæus for that of Von Linné, upon being raiſed to the rank of nobility, as names terminating in *us* are confined to the plebeian order in Sweden.

In 1776 ſoon after his recovery from a ſtroke of apoplexy he drew up a ſhort account of his life, and ſent it to the academy as a part of materials for his eulogy. In this writing, which may be ſtyled the *Confeſſions of Linnæus*, he very candidly acknowledges his foibles. Among other things he allows that he was too eaſily irritated, that he was ſlow in embracing new opinions, and too tenacious of thoſe he had once adopted. He confeſſes alſo, that he was too impatient of criticiſm and contradiction.

In enumerating the honours that have been paid to the memory of Linnæus his eulogiſt ſpeaks of a monument erected by one of his diſciples in the church of Edinburgh. But this is a miſtake, which ſeems to have taken its riſe from an urn having been placed in honour of this great naturaliſt, by Dr. Hope, in the Botanic garden at Edinburgh.

The

The memoirs in this volume, the ſubjects of which are connected with our plan, are as follow:

1. *Remarks on certain ſaline combinations of Iron. By* M. de Laſſone.—The objects of this paper are to aſcertain by experiments; 1ſt, in what manner iron combines, under different circumſtances, with the concrete acid of tartar; 2dly, whether an immediate ſolution of iron can be effected by fixed and volatile alkalis, and what kind of union this metal is capable of contracting with thoſe ſalts.

He finds that after mixing filings of iron and cream of tartar, if the mixture is moiſtened with water and expoſed to a long digeſtion, a combination takes place which is ſoluble in cold water. If tincture of galls is added to this ſolution, ink is formed, but no Pruſſian blue is precipitated by the addition of phlogiſticated alkali, unleſs the liquor is boiled.

M. de Laſſone concludes from this experiment that the martial balls procured by digeſtion are different from thoſe procured by boiling. He gives the preference to the former for medicinal purpoſes.

II. *Second memoir on the Gum tree called Vérek at Senegal, on the manner in which its gum and*

*that of the acacias is collected, and on another tree of the same kind. By* M. Adanſon.—This eſſay is a ſupplement to a former paper by the ſame author on the gum trees of Senegal, printed in the Memoirs of the Academy for 1775.

He here deſcribes two ſpecies of gum trees or *Acacias*; the firſt, called *Uérek* by the natives, is a tree of middle growth which ſeldom exceeds twenty feet in height. Linnæus in his *Species Plantarum*, p. 521, has named it *Mimoſa, Senegal, ſpinis ternis, intermedio reflexo, foliis bipinnatis, floribus ſpicatis.* It yields a white gum, which has a ſweet taſte, combined, when it is freſh, with a ſlight degree of acidity. This gum, we are told, and milk, conſtitute almoſt the whole of the diet of the Arabs who lead a wandering life in the deſerts of Africa. As the gum trees are divided into three great foreſts, ſo theſe people are compoſed of three hordes, each of which has its chief.

The ſecond ſpecies of Acacia deſcribed by our author is much ſmaller than the other. The natives have given it the name of *Ded.* This tree, although of the ſame genus as the Uérek, produces no gum.

III. *An Eſſay on the decompoſition of ſeveral Neutral Salts with baſes of fixed and volatile alka-*

*lis,*

*lis, by the marine acid. By* M. Cornette.—— M. Baumé proved, many years ago, that the nitrous acid decompoſes vitriolated tartar and Glauber's ſalt, and alſo that the vitriolic acid decompoſes both priſmatic nitre and quadrangular nitre. This is a ſpecies of paradox which ſeems to deſtroy, or at leaſt obliges us to modify the doctrine of affinities.

M. Baumé attempted to decompoſe the ſame ſalts by the marine acid, but without ſucceſs.— On the other hand M. Margraff has been able, with the marine acid, to decompoſe the two nitres with fixed alkaline baſes, and even vitriolated tartar and Glauber's ſalt. M. Cornette has undertaken to repeat and extend the experiments of theſe two chemiſts, with a view to reconcile their contradictions, and to throw new lights on the ſubject. In the preſent paper he confines himſelf to the decompoſition of vitriolic and nitrous ſalts with baſes of fixed and volatile alkali, by the marine acid. The acid he employed in his experiments was extremely pure, and care was taken that the ſalts he meant to decompoſe ſhould be in a ſtate of dryneſs. With theſe precautions, each of the above ſalts was decompoſed, but with different circumſtances. The decompoſition of ammoniacal ſalts was was found to be the easiest of all; and next to those, that of salts which have a mineral alkali for their basis. Those with a basis of vegetable alkali were observed to resist decomposition the most.

IV. *Two Essays on the comparative action of the nitrous and marine Acids on vitriolic Salts with an earthy basis. By* the same.——In these two essays M. Cornette continues the inquiry begun in the last paper. He finds that none of the vitriolic salts with an earthy basis are decomposed by the other mineral acids, but, it seems, that either nitrous or marine salts with an earthy basis, decompose the vitriolic salts with an alkaline basis. These are the principal phænomena described by M. Cornette; and altho' they do not overturn the general theory of affinities, yet it must be acknowledged that they destroy the particular laws of affinity which chemists were too eager to adopt, and that they indicate others which ought to be substituted in their stead.

V. *Analysis of the Water of the lake Asphaltites. By* M. M. Macquer, Lavoisier, *and* Sage.——The water of this lake, which is situated in Judæa, is famous for its bitterness, which has been attributed to bitumen, and for its weight. Two bottles of it having been sent to Paris, its weight weight compared with that of diſtilled water was found to be as 5 to 4; a circumſtance not to be equalled by any other ſaline water. It was ſo completely ſaturated that chryſtalliſed ſalt was found at the bottom of the bottles. A quintal of it, we are told, contains about 45 pounds of ſalt, of which $6\frac{1}{2}$ are common marine ſalt, $16\frac{1}{2}$ marine ſalt with an earthy baſis, and 22 marine ſalt with a baſis of the earth of magneſia. The bitumen that iſſues from the bottom of the lake, and floats on its ſurface, gives no impregnation to the water, the bitterneſs of which, like that of ſea water, is owing ſolely to the ſalts it contains.

VI. *Obſervations relative to two Animals, the male of which delivers the female. By* M. Demours.——We have here an account of a ſcene of which the author happened to be an eye witneſs in one of his evening walks in the neighbourhood of Paris. A male frog, of the ſpecies to which Geſner has given the name of *Rubeta minor*, was buſied in delivering his female of her eggs. This curious proceſs is very accurately deſcribed by M. Demours.

VII. *Obſervations on the Red Mine of Copper. By* M. Sage.——This eſſay is intended to prove, that what is called the red mine of copper, is no other other than a calx of copper, or the metal deprived of its phlogiſton.

VIII. *Remarks on the Motion of the Ribs in Reſpiration. By* M. Bordenave.——Theſe remarks tend to prove, that all the ribs are not equally raiſed in inſpiration; that they do not all of them ſeparate from, but, on the contrary, that ſome of them are drawn nearer to each other; that they are moſt elevated anteriorly where their motion is moſt apparent; and that they preſerve nearly the ſame diſtance between each other poſteriorly. M. Bordenave takes occaſion, towards the end of his paper, to point out the ill effects of ſtays and tight lacing in women.

IX. *Caſe of a Fiſtulous Opening in the Abdomen, through which the patient voided almoſt the whole of his urine. By* M. Sabatier.——We have here an account of a patient, whoſe urine being obſtructed, an abſceſs formed near the navel, and diſcharged a conſiderable quantity of pus and urine. This opening, which ſoon became fiſtulous, gradually contracted, and was ſometimes entirely cloſed. When this happened, a freſh ſuppreſſion of urine took place, and the patient experienced ſevere pain, till the diſcharge thro' the fiſtula was reſtored. At length the patient died, and on diſſection, ſeveral ſmall ſtones were found found in the bladder, and one of them at the neck of the bladder obſtructing the urethra. At the upper part of the bladder, was found a communication with the fiſtulous opening in the integuments, by a canal two fingers breadth in length. M. Sabatier has found only two caſes ſimilar to this in books; one of theſe is recorded by Hildanus, and the other in the 3d volume of the Memoirs of the Academy of Surgery.

x. *An Eſſay on the motion of the Ribs, and the action of the intercoſtal Muſcles. By* the ſame.—— The doctrine laid down in this paper is, in many reſpects, ſimilar to that contained in M. Bordenave's. The author obſerves that in inſpiration, the upper ribs only aſcend while the lower ones deſcend, and the middle ones undergo a ſort of rotatory motion from within outwards. As the ribs ſeparate from each other while the cheſt is dilating, the intercoſtals, he remarks, ought no longer to be reckoned amongſt the muſcles that aſſiſt in inſpiration.

xi. *Fourth Memoir on the Anatomy of Birds. By* M. Vicq D'Azyr.——In this paper the ingenious author deſcribes the organ of hearing of birds, and compares it with the ſame organ in man, in quadrupeds, in reptiles, and in fiſh.

 The

The defcriptions are illuftrated by very elegant engravings.

XII. *Extract from Meteorological Obfervations, made in the country near Paris, during the cold weather in January* 1767. *By* M. Adanfon.—— This paper, we are told, is only a fmall part of a very extenfive work on the variations of the atmofphere, on which the author has been employed for more than twenty years paft.

XIII. *Experiments on a fpecies of White Steatites, which is convertible by fire into a fine bifcuit porcelain. By* M. M. Guettard *and* Lavoifier.—— Thefe gentlemen, in a journey they made together through different parts of France, in 1767, difcovered in the neighbourhood of Plombieres a white argillaceous earth, which, without any addition, and even without being wafhed, yields in the fire a fine bifcuit porcelain; a proof that this earth is not a pure clay, but a mixture of clay with a fufible earth.

XIV. *A Defcription of two Coal Mines, fituated at the foot of the Mountains of Voyes, one in Franche Comté, the other in Alface, with fome experiments on the coal they contain. By* the fame.

XV. *An Account of a new Method of affaying Gold. By* M. Tillet.

XVI. *General Remarks on the nature of Acids, and on the principles of which they are composed. By* M. Lavoisier.——In different papers printed in former volumes of the Memoirs of the Academy, the learned author thinks he has clearly proved, that dephlogisticated air enters as a constituent part into the composition of several acids, and particularly of the phosphoric, vitriolic, and nitrous. Farther experiments have enabled him, he tells us, to assert, that this same species of air is the constituent principle of acidity; that it is, of course, common to every acid; and that it is the presence of one or more other principles along with it that constitutes the different species of acids. Thus, for example, combined with sulphur, it forms vitriolic acid; with nitrous air, the nitrous acid; with phosphorus, the phosphoric acid; and with sugar, the acid of sugar.

This acidifying principle, *(principe acidifiant)* as he calls it, combined with metallic substances, forms, we are told, with the greater number of them a metallic calx; but some of these substances, as arsenic, iron, and perhaps several others, being combined with the acidifying principle to a certain degree of superabundance, assume not only a saline character, but even acquire the properties common to acids, and like them become true folvents.

M. Lavoifier remarks alfo; 1ft, that this principle has, like every other, its different degrees of affinity; that it has, for example, a much greater affinity with fugar, and with the generality of vegetable and animal fubftances, than with nitrous air; and that in confequence of this preference, it quits the latter to form with thofe fubftances different fpecies of acids: 2dly, that the number of acids that may be formed is as yet abfolutely undetermined, as we are not yet acquainted with all the fubftances that are fufceptible of combining with the acidifying principle, and are ftill lefs acquainted with the means to be employed for attaining fuch a combination.

XVII. *Report made to the Academy concerning the Gold which may be procured from Vegetable Earths or Afhes.*—It feems that in 1778 M. Sage gave an account to the Academy of fome experiments, from which it appeared that a quintal of common garden earth, upon being combined with a certain proportion of minium, and calcined, had yielded two ounces and forty-four grains of gold. The Comte de Lauraguais having repeated M. Sage's experiments, and found found the refult of them very different from what that chemift had afferted, requefted the Academy to appoint a Committee who might clear up the point in difpute. Meffieurs Macquer, Cadet, Lavoifier, Baumé, Bucquet, and Cornette, were accordingly nominated for this purpofe. Thefe celebrated chemifts give a very fatisfactory account of their experiments, and the refult is, that the quantity of gold procured by combining minium with afhes is infinitely fmall, not more than one or two grains from a quintal; and they are convinced that the greater part, if not the whole of this fmall quantity of gold exifted in the minium; and they think it probable that the minium employed by M. Sage in his experiments, contained a greater proportion of gold than the minium does which is commonly met with in trade.

XVIII. *Botanico-Meteorological Obfervations made at the Caftle of Denainvilliers, near Pithiviers in Gatinois, in* 1777. *By* M. Du Hamel.

XIX. *A Mineralogical Effay. By* M. Montet.— This paper relates to the mineralogy of fome parts of Languedoc.

