# Errata

**Published:** 1783

**Authors:** 


					ERRATA.
Page 26, line 3 from the top, for Volmue read Volume ;

				

